# Updates to the checklist of the wild bee fauna of Luxembourg as inferred from revised natural history collection data and fieldwork

**DOI:** 10.3897/BDJ.9.e64027

**Published:** 2021-05-14

**Authors:** Fernanda Herrera Mesías, Alexander M. Weigand

**Affiliations:** 1 Department of Animal Ecology, Evolution and Biodiversity, Ruhr-Universität Bochum, Bochum, Germany Department of Animal Ecology, Evolution and Biodiversity, Ruhr-Universität Bochum Bochum Germany; 2 Musée national d’histoire naturelle de Luxembourg, Luxembourg, Luxembourg Musée national d’histoire naturelle de Luxembourg Luxembourg Luxembourg

**Keywords:** biological collections, Apoidea, taxonomy, DNA barcodes, Benelux

## Abstract

**Background:**

Museums and other institutions curating natural history collections (NHCs) are fundamental entities to many scientific disciplines, as they house data and reference material for varied research projects. As such, biological specimens preserved in NHCs represent accessible physical records of the living world's history. They provide useful information regarding the presence and distribution of different taxonomic groups through space and time. Despite the importance of biological museum specimens, their potential to answer scientific questions, pertinent to the necessities of our current historical context, is often under-explored.

The currently-known wild bee fauna of Luxembourg comprises 341 registered species distributed amongst 38 different genera. However, specimens stored in the archives of local NHCs represent an untapped resource to update taxonomic lists, including potentially overlooked findings relevant to the development of national conservation strategies.

**New information:**

We re-investigated the wild bee collection of the Zoology Department of the National Museum of Natural History Luxembourg by using morphotaxonomy and DNA barcoding. The collection revision led to the discovery of four species so far not described for the country: *Andrena lagopus* (Latreille, 1809), *Nomada furva* (Panzer, 1798), *Hoplitis papaveris* (Latreille, 1799) and *Sphecodes majalis* (Pérez, 1903). Additionally, the presence of *Nomada sexfasciata* (Panzer, 1799), which inexplicably had been omitted by the most current species list, can be re-confirmed. Altogether, our findings increase the number of recorded wild bee species in Luxembourg to 346. Moreover, the results highlight the crucial role of NHCs as repositories of our knowledge of the natural world.

## Introduction

Natural history museums are important research and educational institutions, with a crucial role in the production and communication of scientific knowledge. Their associated natural history collections (NHCs) document what is known about the world’s bio- and geodiversity, provide resources and reference material for scientific research and outreach and contribute to the very basis of formal educational programmes (Lane 1996, Pyke and Ehrlich 2010, Bradley et al. 2014, Kharouba et al. 2018). Archived biological museum specimens provide valuable sources of data to many different kinds of research projects, with genetic and phylogenetic information being present as an inherent part the organisms themselves, while ecological and biographical information can be retrieved from their associated metadata (Lane 1996). Moreover, museum data constitute a rich source of historical records of species occurrences, documenting changes in the presence (and potential absence) of organisms. This kind of information has a central role in conservation biology initiatives (Shaffer et al. 1998). As such, museum samples and their records represent an untapped resource of knowledge that can complement the data retrieved by past and ongoing biological surveys (Lane 1996, Lister 2011). The strong and weak points of NHCs data (i.e. extended timeframe, but patchy temporal coverage) complement those of data retrieved from contemporary monitoring studies (i.e. detailed temporal information, but within a short timeframe), thus improving the predictive power of integrative studies (Kharouba et al. 2018). With this approach in mind, historical museum records can be used: i) as a reference to study species’ range shifts (Kharouba et al. 2018), ii) to identify population declines (Shaffer et al. 1998) and iii) in the assembly and update of taxonomically focused species inventories. Finally, since NHCs not just provide verifiable spatio-temporal references, but the preserved specimens themselves, they allow the re-examination of both - data and voucher - to validate scientific knowledge in the light of new technological advances or discoveries (Monfils et al. 2017, Kharouba et al. 2018).

In this study, we followed this approach and performed a revision of the wild bee collection material curated at the Zoology Department of the National Museum of Natural History Luxembourg (Musée national d'histoire naturelle de Luxembourg; MNHNL) to produce an updated species checklist of the wild bee fauna of Luxembourg. Additionally, specimens collected in 2019 during the pilot phase of the ongoing "Atlas of the wild bee fauna of Luxembourg” project were integrated in the analysis to evaluate the presence of potential new findings.

## Materials and methods

Relevant entries of 5,908 wild bee voucher specimens stored in the database of the MNHNL (accession number, preferred determination and gathering site) were downloaded as spreadsheets to evaluate the presence and distribution of Luxembourgish and non-Luxembourgish species in the collection (GBIF 2021). Specimen annotations were compared against a newly-compiled species checklist, based on the species records listed in Rasmont et al. (2017), Vereecken (2018), Schneider (2018) and Weigand and Herrera-Mesías (2020), which correspond to the most recent literature reporting wild bee species for the country. All together, an initial reference list with 341 species was considered for the Luxembourgish wild bee fauna. The European Red List of bees (Nieto et al. 2014) was used to determine the conservation status of the species.

Spreadsheets were manipulated using R version 3.6.2 (R Core Team 2019) to identify mismatches between the species described in the museum records and the species mentioned in the publications. In case of mismatch, the registered gathering sites of the collection specimens were checked to evaluate if they were located in the country. Specimens fulfilling both criteria, according to the information on their labels (i.e. collected in Luxembourg, but corresponding to a species not described as currently being present in the country), were physically retrieved from the collection.

From the pilot phase of the wild bee atlas project, 16 specimens were selected for molecular identification via DNA barcoding. The BF2/BR2 primer pair (Elbrecht and Leese 2017) was used to amplify a 421 bp region within the mitochondrial cytochrome c oxidase subunit I gene (COI), which is the most frequently investigated genetic marker gene for barcoding animals. The laboratory protocols of Weigand and Herrera-Mesías (2020) for DNA extraction, polymerase chain reaction (PCR), PCR purification and Sanger sequencing were used. However, the PCR thermal cycling was based on the temperatures described in Elbrecht and Steinke (2019). The PCR started with an initial denaturation step at 94°C for 5 minutes, followed by 34 cycles of denaturation for 30 seconds at 94°C with annealing for 30 seconds at 50°C and extension at 65°C for 50 seconds; and a final extension for 5 minutes at 65°C. The produced chromatograms were visually inspected and edited using Geneious Prime 2019.1.1 (Kearse et al. 2012). The individual COI DNA barcodes were compared against sequences stored in the Barcode of Life Data system (BOLD; Ratnasingham and Hebert 2007). The annotations suggested by the molecular results were then evaluated against the local species list from literature.

Potential new species discoveries originating from either the revised museum collection entries or the fieldwork material were inspected with a Keyence VHX-S660E digital microscope, using various morphological keys to evaluate diagnostic traits (Table 1).

## Taxon treatments

### Nomada furva

(Panzer, 1798)

11409551-665C-5A11-98D1-1B559156557B

Nomada furva Common names: Nomade funeste (French), Schwärzliche Wespenbiene (German)

#### Materials

- **Type status:** Other material. **Occurrence:** catalogNumber: MNHNL39915; recordedBy: Fernand Feitz; sex: male; lifeStage: adult; **Taxon:** scientificName: *Nomada furva* (Panzer, 1798); order: Hymenoptera; family: Apidae; genus: Nomada; specificEpithet: *furva*; taxonRank: species; scientificNameAuthorship: (Panzer, 1798); vernacularName: Nomade funeste (French), Schwärzliche Wespenbiene (German); **Location:** country: Luxembourg; locality: Remerschen; decimalLatitude: 49.4837; decimalLongitude: 6.3475; **Identification:** identifiedBy: Andrea Jakubzik; **Event:** samplingProtocol: Net; eventDate: 16-07-2004; **Record Level:** institutionCode: MNHNL; basisOfRecord: Preserved Specimen

#### Diagnosis

**Figure 1.**
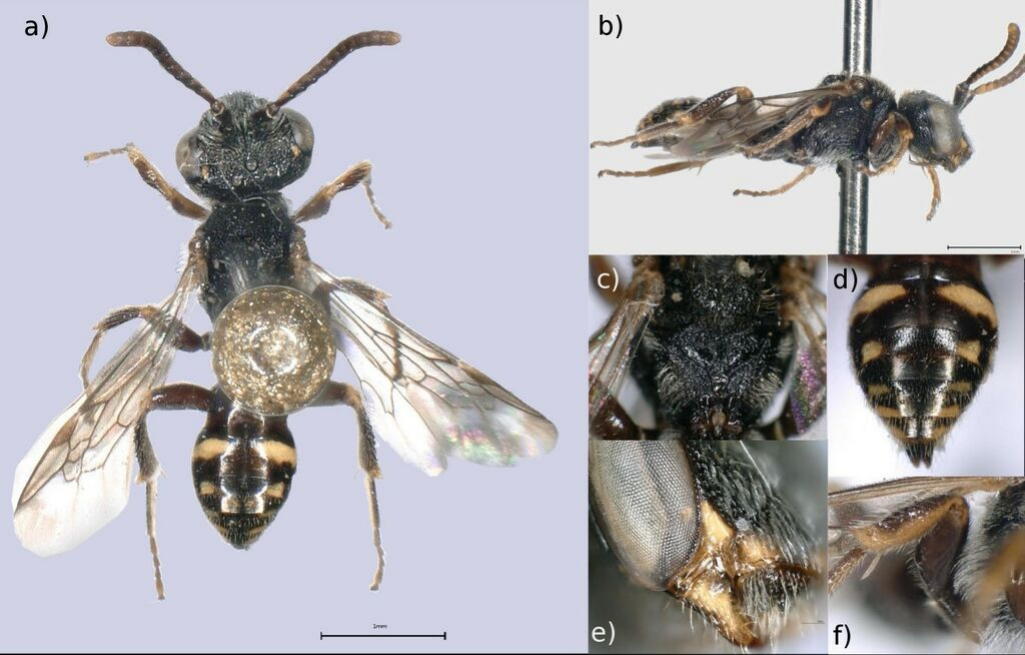
*Nomada furva* (Panzer, 1798) (Remerschen, specimen MNHNL39915). **a.** Dorsal view (scale bar = 1 mm); **b.** Lateral view (scale bar = 1 mm); **c.** Propodeum, showing tuft of pale hair; **d.** Abdomen, yellow spots/bands and notched tergite 7; **e.** Labrum tooth (scale bar = 100 µm); **f.** Femur 2. Photos: MNHNL. Background edited using GIMP 2.8.22 (The GIMP Development Team 2020).

Male: A small (4-6 mm) dark coloured *Nomada* presenting yellow maculations in the margin of labrum, malar area, apex of clypeus and in front of the eyes (Fig. 1a and b). The lower part of the sides of the propodeum shows a small sub-erect tuft of pale hair (Fig. 1c). The dark brown abdomen presents impuctated tergites with lateral yellow spots and a notched tergite 7 (Fig. 1d). The labrum presents a small tooth (Fig. 1e) and the femur 2 is widened (Fig. 1f).

Voucher specimen (MNHNL39915): Adult male. Body length of 4.7 mm.

#### Ecology

A bivoltine species that parasites the nests of small *Lasioglossum*, with a flight season that extends from the end of April until mid-September (Amiet et al. 2007).

#### Conservation

*Nomada furva* is classified under the IUCN category “Data Deficient (DD)”, meaning that there is a lack of scientific information to assess extinction risk (Nieto et al. 2014). More information regarding its population size, distribution, trends and potential threats to this species is needed (Smit 2013).

### Hoplitis papaveris

(Latreille, 1799)

67382451-6A9B-5F81-8BA9-F874E30E9C0F

Hoplitis papaveris Common names: Osmie du Coquelicot (French), Mohn-Mauerbiene (German).

#### Materials

- **Type status:** Other material. **Occurrence:** catalogNumber: MNHNL21866; recordedBy: Svenja Christian; sex: male; lifeStage: adult; **Taxon:** scientificName: *Hoplitis papaveris* (Latreille, 1799); order: Hymenoptera; family: Megachilidae; genus: Hoplitis; specificEpithet: *papaveris*; taxonRank: species; scientificNameAuthorship: (Latreille, 1799); vernacularName: Osmie du Coquelicot (French), Mohn-Mauerbiene (German); **Location:** country: Luxembourg; locality: Mertert; decimalLatitude: 49.70359; decimalLongitude: 6.48171; **Event:** samplingProtocol: Caught by hand; eventDate: 04-06-2015; **Record Level:** institutionCode: MNHNL; basisOfRecord: Preserved Specimen

#### Diagnosis

**Figure 2.**
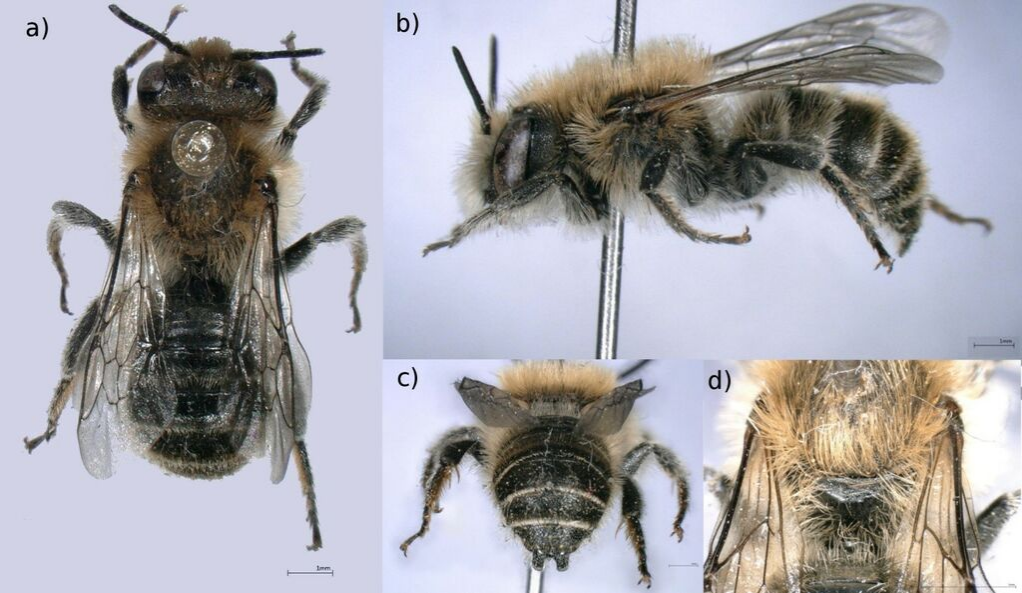
*Hoplitis papaveris* (Latreille, 1799) (Mertert, specimen MNHNL21866). **a.** Dorsal view; **b.** Lateral view showing abdomen curvature; **c.** Abdomen, showing the two terminal lobules of tergite 7; **d.** Detailed view of the propodeum (shiny surface). Scale bars = 1 mm. Photos: MNHNL. Background edited using GIMP 2.8.22 (The GIMP Development Team 2020).

Male: A medium sized (9-11 mm) mason bee with a black body covered in yellow-brown hair and short antennae (Fig. 2a). Tergite 6 is strongly curved (Fig. 2b) and tergite 7 presents a characteristic forked edge (Fig. 2c). The midfield part of the propodeum has a polished terminal surface (Fig. 2d).

Voucher specimen (MNHNL21866): Adult male. Body length of 8.8 mm.

#### Ecology

A ground-nesting bee that builds its nest in sandy soil, using pieces of poppy petals (*Papaver rhoeas*) to line the brood cells (Günter 1997, Amiet et al. 2004). Its flight season spans from late May to mid July (Amiet et al. 2004).

#### Conservation

*Hoplitis papaveris* is classified under the IUCN category “Least Concern (LC)” (Nieto et al. 2014), but it is listed in the National Red Lists and Red Data Books of the Czech Republic, Germany, the Netherlands and Switzerland, emphasising that further research is needed regarding the species population sizes, trends and threats (Lhomme 2014).

### Nomada sexfasciata

(Panzer, 1799)

61DAE441-E835-587B-8939-D61C404ED6DC

Nomada sexfasciata Common names: Nomade six-bandes (French), Langkopf-Wespenbiene (German), Six-banded Nomad Bee (English).

#### Materials

- **Type status:** Other material. **Occurrence:** catalogNumber: MNHNL25076; recordedBy: Fernand Feitz; sex: female; lifeStage: adult; **Taxon:** scientificName: *Nomada sexfasciata* (Panzer, 1799); order: Hymenoptera; family: Apidae; genus: Nomada; specificEpithet: *sexfasciata*; taxonRank: species; scientificNameAuthorship: (Panzer, 1799); vernacularName: Nomade six-bandes (French), Langkopf-Wespenbiene (German), Six-banded Nomad Bee (English); **Location:** country: Luxembourg; locality: Remich; decimalLatitude: 49.5479; decimalLongitude: 6.36037; **Identification:** identifiedBy: Andrea Jakubzik; **Event:** samplingProtocol: Net; eventDate: 05-05-2000; **Record Level:** institutionCode: MNHNL; basisOfRecord: Preserved Specimen

#### Diagnosis

**Figure 3.**
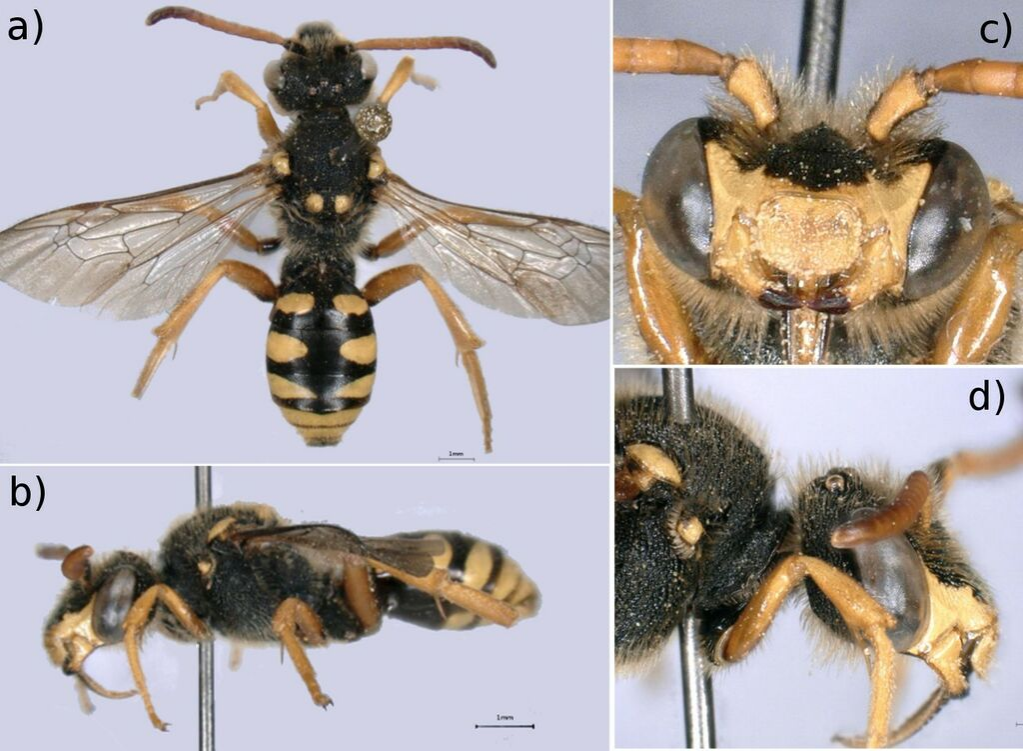
*Nomada sexfasciata* (Panzer, 1799) (Remich, specimen MNHNL25076). **a.** Dorsal view; **b.** Lateral view; **c.** Face, frontal view showing maculations, mandibles, clypeus, labrum and longitudinal carina; **d.** Face, lateral view showing elongated malar area. Scale bars = 1 mm. Photos: MNHNL. Background edited using GIMP 2.8.22 (The GIMP Development Team 2020).

Female: A large (11-13 mm) black and yellow Nomada species. Yellow maculations are present in the pronotal lobes, tegula, as two small spots on the scutellum and as large yellow spots on the sides of tergites 1-3, that become bands in tergites 4-5 (Fig. 3a and b). The head presents a sharp longitudinal carina between the antennae (Fig. 3c). Yellow maculations are present on the mandibles (apex simple), the clypeus and labrum, the malar area, the para-ocular area and on the ventral surface of the scape (Fig. 3c). The malar area is characteristically long with a protruding lower face (Fig. 3d) and the labrum is rounded, without a tooth (Fig. 3e).

Voucher specimen (MNHNL25076): Adult female. Body length of 12 mm.

#### Ecology

An univoltine species that parasites *Eucera* nests, with a flight season spanning from late April until late July on the European continent (Amiet et al. 2007).

#### Conservation

*Nomada sexfasciata* is classified under the IUCN category “Least Concern (LC)” (Nieto et al. 2014), but it is included in the National Red Lists and Red Data Books of Great Britain, the Netherlands and Sweden (Smit 2014).

### Andrena lagopus

(Latreille, 1809)

F86547C4-1DF0-5E0D-BFBD-11E2FF45A4DB

Andrena lagopus Common names: Andrène deux-cellules (French), Zweizellige Sandbiene (German).

#### Materials

- **Type status:** Other material. **Occurrence:** catalogNumber: MNHNL100056; sex: female; lifeStage: adult; associatedSequences: BOLD: MNHNL162-21; **Taxon:** scientificName: *Andrena lagopus* (Latreille, 1809); order: Hymenoptera; family: Andrenidae; genus: Andrena; specificEpithet: *lagopus*; taxonRank: species; scientificNameAuthorship: (Latreille, 1809); vernacularName: Andrène deux-cellules (French), Zweizellige Sandbiene (German); **Location:** country: Luxembourg; locality: Kehlen; decimalLatitude: 49.671931; decimalLongitude: 6.046718; **Identification:** identifiedBy: Fernanda Herrera-Mesías; **Event:** samplingProtocol: pan trap (passive sampling); eventDate: 20-05-2019; **Record Level:** institutionCode: MNHNL; basisOfRecord: Preserved Specimen

#### Diagnosis

**Figure 4.**
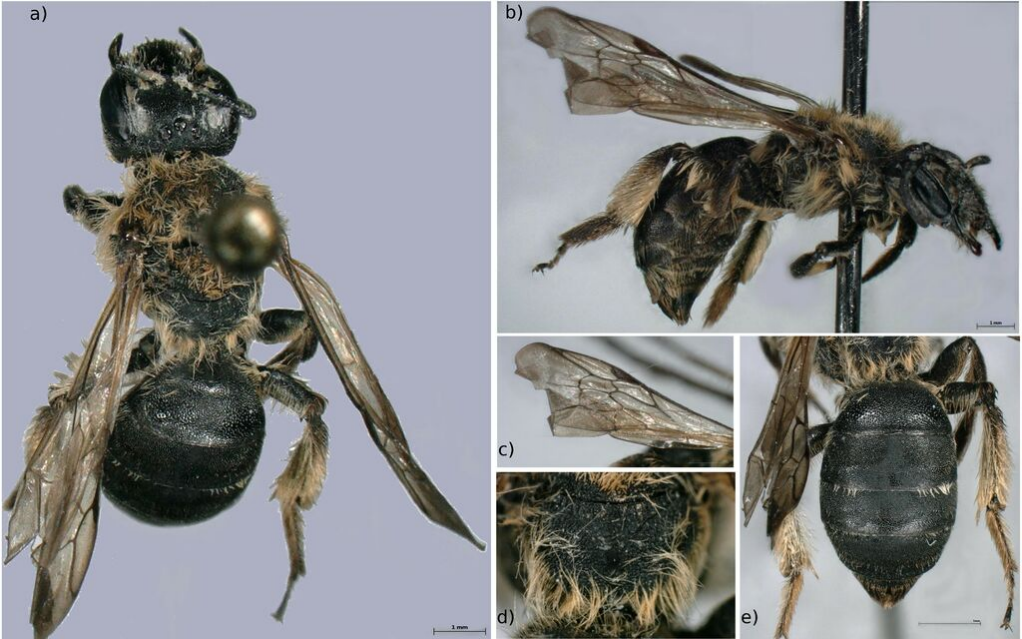
*Andrena lagopus* (Latreille, 1809) (Kehlen, specimen MNHNL100056; BOLD identifier MNHNL162-21, one middle leg was used for DNA extraction). **a.** Dorsal view; **b.** Lateral view; **c.** Wing, showing submarginal cells; **d.** Propodeum; **e.** Abdomen, showing band and punctures in the tergites. Photos: MNHNL. Background edited using GIMP 2.8.22 (The GIMP Development Team 2020).

Female: A medium size ground nesting bee with brownish hair and yellowish scopae (Fig. 4a and b). This species is characterised by having only 2 submarginal cells, despite being an *Andrena* (Fig. 4c). The middle part of the propodeum is coarsely wrinkled, clearly defined against the sides (Fig. 4d). The abdomen presents scarce, interrupted hair bands and densely punctured tergites, with very narrow spaces amongst the punctures on the surface of tergite 2 (Fig. 4e).

Specimen from pilot study (MNHNL100056; BOLD identifier MNHNL162-21): Adult female. Body length of 9.23 mm.

##### Molecular identification

The taxonomic annotation is supported by DNA barcoding data. The best percentages of sequence identity were achieved with two specimens of *Andrena lagopus* from France (100% each, BOLD identifiers FBHAP970-09 and POLLE2072-19), collected in Alsace and Indre et Loire.

#### Ecology

A univoltine species, that specialises on crucifers (Brassicaceae) for pollen collection (Amiet et al. 2010). Its flight season spans from early April until mid-June in Europe (Amiet et al. 2010).

#### Conservation

*Andrena lagopus* is regionally classified as “Least Concern (LC)” (Nieto et al. 2014) as it is widespread across much of western and central Europe, being recorded in nearby countries, such as France, Germany and Switzerland (Roberts and Meulemeester 2015, Rasmont et al. 2017).

### Sphecodes majalis

(Pérez, 1903)

A68A4ADA-245F-5A6A-8572-B7C6269E7294

Sphecodes majalis Common names: Sphécode de mai (French), Kortsnuitbloedbij (Dutch), Mai-Blutbiene (German).

#### Materials

- **Type status:** Other material. **Occurrence:** catalogNumber: MNHNL100057; sex: male; lifeStage: adult; associatedSequences: BOLD: MNHNL163-21; **Taxon:** scientificName: *Sphecodes majalis* (Pérez, 1903); order: Hymenoptera; family: Halictidae; genus: Sphecodes; specificEpithet: *majalis*; taxonRank: species; scientificNameAuthorship: (Pérez, 1903); vernacularName: Sphécode de mai (French), Kortsnuitbloedbij (Dutch), Mai-Blutbiene (German); **Location:** country: Luxembourg; locality: Manternach; decimalLatitude: 49.710039; decimalLongitude: 6.430916; **Identification:** identifiedBy: Fernanda Herrera-Mesías; **Event:** samplingProtocol: pan trap (passive sampling); eventDate: 28-04-2019; **Record Level:** institutionCode: MNHNL; basisOfRecord: Preserved Specimen

#### Diagnosis

**Figure 5.**
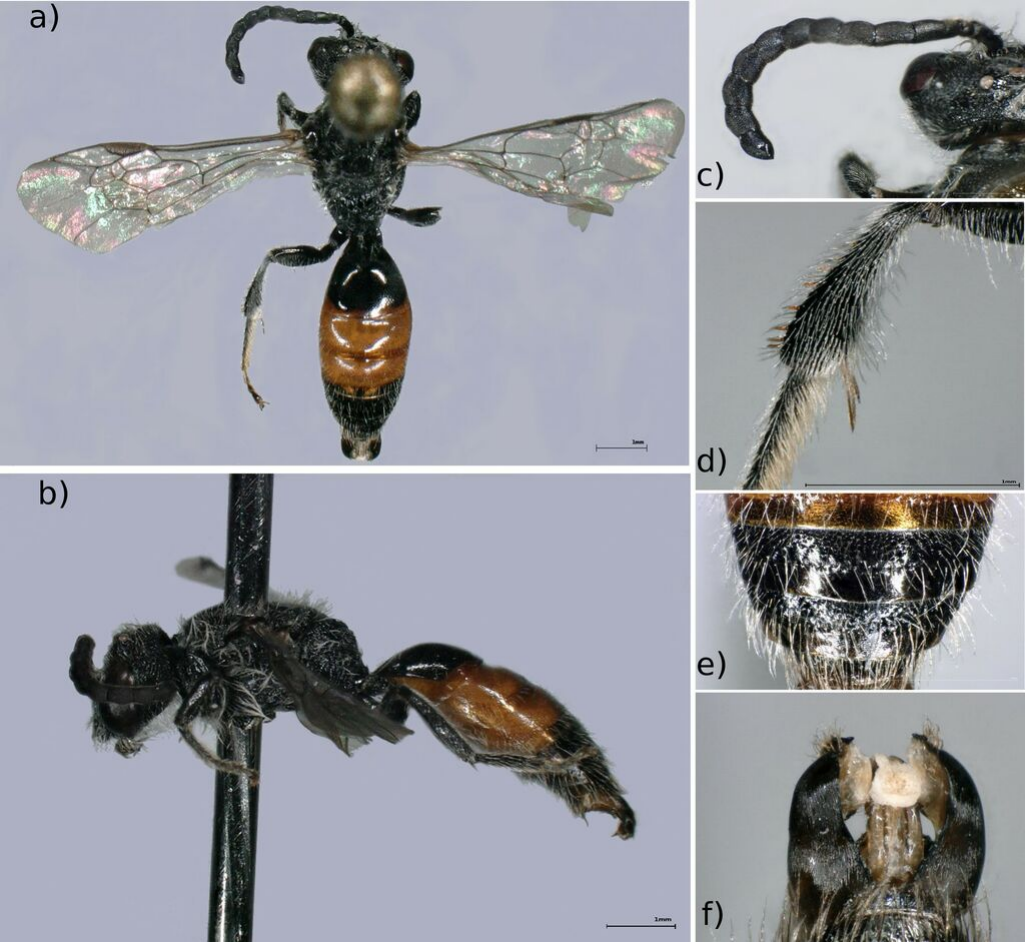
*Sphecodes majalis* (Pérez, 1903) (Manternach, specimen MNHNL100057; BOLD identifier MNHNL163-21, one middle leg was used for DNA extraction). **a.** Dorsal view; **b.** Lateral view; **c.** Antenna, showing flagellar segments; **d.** Left hind tibia, showing spines in pale pubescence; **e.** Tergites 4 and 5; **f.** Genitalia. Photos: MNHNL. Background edited using GIMP 2.8.22 (The GIMP Development Team 2020).

Male: A medium sized *Sphecodes* species (6-8 mm) (Fig. 5a and b). Flagellar segments of the antenna longer than broad, without pubescence in the front side (Fig. 5c). The hind tibia present red spines on its upper side amongst a covering of pale hairs (Fig. 5d). The punctures on tergites 4 and 5 are subtle and very sparsely distributed, presenting fine sculpture amongst them (Fig. 5e). The gonocoxites lack any impression and the gonostyli have a rounded edge (Fig. 5f).

Voucher specimen (MNHNL100057; BOLD identifier MNHNL163-21): Adult male. Body length of 7.19 mm.

##### Molecular identification

The taxonomic annotation is supported by DNA barcoding data. A perfect match of genetic sequence similarity was achieved with a specimen of *Sphecodes majalis* from France (100%; BOLD identifier POLLE1165-19), collected in Loir et Cher.

#### Ecology

A rare cuckoo bee that inhabits steppes and sunny sites, flying only during a short time span in spring from late March until mid-May, matching the flight season of *Lasioglossum pallens* (Brullé, 1832), its only known host in Europe (Amiet et al. 2007,Bogusch and Straka 2012).

#### Conservation

*Sphecodes majalis* is classified as “Near Threatened (NT)” (Nieto et al. 2014). Records of this bee and its host are scarce and the populations of both are described as locally decreasing (Bogusch and Straka 2014).

## Analysis

### Annotations and potential new species records

The 5,908 wild bee specimens registered in the MNHNL collection were distributed amongst 33 genera from six families (Andrenidae, Apidae, Colletidae, Halictidae, Megachilidae and Melittidae). Exact sampling sites and detailed information for each museum specimen are available at the Species Observation Database Service of the museum. From the 218 wild bee species listed in the database, 194 were present in previous species checklists, confirming that at least some individuals have been found in Luxembourgish territory. The remaining 24 species did not figure as present in the country in the literature sources consulted. These species were represented in the collection exclusively by individuals collected in other countries, except for five species, whose annotations linked them to at least one voucher specimen from a gathering site placed in modern Luxembourgish territory: *Colletes fodiens* (Fourcroy, 1785), *Hylaeus styriacus* (Förster, 1871), *Nomada furva* (Panzer, 1798), *Nomada sexfasciata* (Panzer, 1799) and *Hoplitis papaveris* (Latreille, 1799). In three of these cases, previous taxonomic annotations of the analysed voucher specimens and our morphological re-examinations were in agreement (for *N. furva*, *N. sexfasciata* and *H. papaveris*).

However, in the remaining two cases, the taxonomic annotations showed conflicting results. In the case of *C. fodiens*, there were two registered specimens (one male and one female) in the collection. The results of the morphological re-evaluation of both specimens were inconclusive. The female specimen (MNHNL41840), which was collected in 1999, lacked the conspicuous white-haired face and those hairs across the dorsal surface of the first tergite that characterise *C. fodiens* (sensu Amiet et al. 1999, Falk and Lewington 2015 and Falk and Lewington 2015). Some authors mention that *C. fodiens* females have a tendency to lose hair as they age, which might make them resemble *C. similis* females (Falk and Lewington 2015). However, without these traits, *C. similis*, as a potential annotation, cannot be excluded. The male specimen (MNHNL41841), which was collected in 2013, had the hairs of the sternites glued together and partially covered by sand grains. The genitalia were contracted inside the abdomen, obscuring visualisation of the crucial characters described in the taxonomic keys. As a more conservative approach, we thus do not recognise *C. fodiens* as a potential new finding for Luxembourg.

In the case of *H. styriacus*, there were eleven female specimens in the collection. In all cases, the shape of the facial fovea matched the one of *Hylaeus* from the subgenus Paraprosopis (sensu Dathe et al. 2016) to which *H. styriacus* belongs. However, the morphological re-evaluation of the voucher specimens suggested different annotations. One of the specimens (MNHNL41982) presented the tridentate mandible and pronotum with pointed edges that characterise *Hylaeus clypearis* (Schenck, 1853). In the rest of the cases evaluated, the characteristics of the specimens matched the description of *Hylaeus pictipes* (Nylander, 1852): facial lateral yellow spots, punctures on tergites/mesopleura and the overall shape of the head. For the aforementioned reasons, the original annotation and the presence of *H. styriacus* in Luxembourg remains to be confirmed.

Finally, the DNA barcodes of two of the specimens from the pilot phase of the wild bee atlas project had a 100% genetic similarity in BOLD with sequences annotated as *Andrena lagopus* (Latreille, 1809) and *Sphecodes majalis* (Pérez, 1903), respectively. Both species have no previous records in the country, rendering those two entries the first documented findings.

Therefore, the morphological and molecular evidence indicates the presence of five wild bee species that are missing in the most current checklists (Suppl. material 1)

## Discussion

The taxonomic re-evaluation of the wild bee collection material curated at the MNHNL, together with newly-collected specimens from the pilot phase of an ongoing atlas project, revealed the presence of five additional wild bee species for Luxembourg. The geographic information stored in the records of the voucher specimens confirmed that at least some individuals have been found on modern Luxembourgish territory, during sampling campaigns performed over the last 20 years. Four out of these five species have not been described as present in the country in any previous publication (*Andrena lagopus*, *Nomada furva*, *Hoplitis papaveris* and *Sphecodes majalis*). The fifth species, *Nomada sexfasciata*, is missing in Rasmont et al. (2017), despite being described as present in Belgium, Switzerland and France by the same authors. Interestingly, an older checklist, available in Rasmont et al. (1995), registered *N. sexfasciata* for Luxembourg as well. Moreover, two specimens (one male and one female) were collected in the country in 1997 (Feitz et al. 2006). The investigated museum material indicates that *N. sexfasciata* was collected in Luxembourg until the year 2009. Hence, we re-added this species to the updated list of Luxembourgish wild bee species.

Additionally, during database cross-checking, we discovered 15 occurrence records on GBIF of *Stelis minuta* Lepeletier & Audinet-Serville, 1825 (Slieker et al. 2021), placed in the national territory. However, those specimens are housed in the Natural History Museum Rotterdam (the Netherlands) and were collected in August 1965 and August 1968 at Rodershausen (50.03N, 6.12E), close to the river Our. This species is not mentioned as present in Luxembourg in all literature sources consulted and no specimens exist in the collection of the MNHNL. Therefore, given that the only records of *S. minuta* in the country are more than 50 years old, as well as the fact that we have not yet been able to physically cross-validate the aforementioned museum specimens, *S. minuta* is not considered in the updated species list.

Furthermore, the Checklist of the Western Palaearctic Bees (Kuhlmann et al. 2021) suggested another seven wild bee species for Luxembourg that are absent in all the literature sources consulted. However, the records of these species corresponded to either wrongly encoded database entries (e.g. stated for the Grand Duchy of Luxembourg, but in reality, the records came from the Belgian Province that is also called "Luxembourg") or cases in which it was just not possible to physically cross-validate the specimens and their geographical information (Suppl. material 2). Therefore, they were also excluded from the species list.

Taken together, our validated findings raise the number of registered wild bee species in Luxembourg from 341 to 346 (Suppl. material 1).

### Discrepancies between current morphological assessment and registered taxonomic annotations of collection specimens

Although, in most cases, the visual inspection of the collection specimens confirmed the results of previous taxonomic assignments, there were two instances of disagreement in which the original taxonomic annotation could not be confirmed with our morphological re-evaluation.

In the case of the potential *C. fodiens* registered in the collection, traditional taxonomic techniques did not allow us to confirm the suggested annotation due to unfavourable specimen conditions. Even though the morphological assessment provided inconclusive results, molecular taxonomic tools might be able to provide further information regarding the identity of similar *Colletes* specimens. Given that the two closely-related species *C. fodiens* and *C. similis* have an estimated genetic distance of 2.17% (Schmidt et al. 2015), DNA barcoding may be effective to separate the two taxa in fresh specimens. Regarding the *Hylaeus* individuals which were originally described as *H. styriacus*, the morphological keys, used in the re-evaluation, suggested two different annotations for the bees stored in the collection (*H. clypearis* and *H. pictipes*). It is worth mentioning that the most important morphological traits used to separate species within the subgenus Paraprosopis, according to the key of Dathe et al. (2016), could only be observed by using a large-scale free-angle observation system, which outperforms the capacity of a common light microscope. Due to the size of the *Hylaeus* bees (~ 4 mm), distinguishing similar species without such a level of magnification and optical performance might not be straightforward, as it may be hard to observe the most subtle details in the head and face. The importance of this factor as a potential error source in the morphological identification of *Hylaeus* bees and other wild bees of similar size remains to be determined.

### *Andrena lagopus* and *Sphecodes majalis*: ecological remarks and local importance

The two new findings of *Andrena lagopus* and *Sphecodes majalis* require a special ecological discussion. Both were collected in 2019 during the pilot phase of a wild bee atlas project. In particular, the discovery of the ground nesting bee, *A. lagopus*, is locally relevant. This species is described as an oligolectic, warm-habitat-loving bee with an Atlanto-Mediterranean distribution (Westrich and Schwenninger 1997, Zettel et al. 2019). Given its specialised preference for pollen sources from the family Brassicaceae, the dispersal and distribution of this species is strongly dependent on the presence of its plant hosts, such as *Brassica napus* (most common pollen source), *Sinapsis arvensis*, *Barbarea vulgaris* and *Cardamine pratensis* (Westrich and Schwenninger 1997). Although the foraging habitats of this bee most commonly include rapeseed fields, set-aside arable fields, ruderal sites, orchards, meadows and gardens, *A. lagopus* has been described as a pioneer species with a high capacity for colonising new foraging habitats (Westrich and Schwenninger 1997). Once considered an endangered species in Germany, it is hypothesised that the large-scale cultivation of rapeseed might have contributed to its recovery and spread (Westrich and Schwenninger 1997, Zettel et al. 2019). The Luxembourgish specimen was found in a meadow in Kehlen, where plants from the Brassicaceae family might be available nearby. Moreover, the specimen presented fully loaded scopae when it was collected, indicating that foraging activity took place shortly before sampling, probably in the surrounding area. Further research would be necessary to determine whether the newly-recorded presence of *A. lagopus* in Luxembourg is related to a recent expansion of its plant hosts. As the pollen loads were collected and preserved in glycerol, future pollen analysis might provide further information on this matter.

Another interesting finding comprises the discovery of *S. majalis* in an orchard in Manternach. This cuckoo bee has a Western Palaearctic distribution that extends across Spain, Algeria, Belgium, southern France, Italy, Hungary, Romania, Switzerland, southern Ukraine, Croatia and The Netherlands, with a potential subspecies (*Sphecodes majalis barbatus*) in Turkey (Warncke 1992, Raemakers 2004). It is considered one of the rarest blood bees in Central Europe and very little is known about its biological cycle (Herrmann et al. 2003, Raemakers 2004, Streese 2020). Available information suggests that its life cycle and parasite-host relationship might be quite unique: it is a species-specific brood parasite of *Lasioglossum pallens*, a warm-climate-loving sweat bee with a preference for trees as pollen sources (Herrmann et al. 2003). It has been speculated that the narrow flight season of *S. majalis* (males typically fly from mid-April to early May and females from mid-April to late May) might be a consequence of the particular relationship this cuckoo bee has with its host. Unlike most other blood bees, which visit different nests and kill or expel their occupants, it has been observed that *S. majalis* females do not drive away its host, showing no aggressive or defensive behaviour towards *L. pallens* females (Herrmann et al. 2003). As the cuckoo bees seem to be tolerated by its hosts, it is possible that the female *S. majalis* remains in the nest for much longer periods than other blood bees, reducing the chances of spotting them in the field (Herrmann et al. 2003). Most likely, this high level of specialisation constrains the distribution of *S. majalis* to the occurrence of its host bee, as well as the types of habitat in which it can be found. So far, *L. pallens* has not been reported in Luxembourg, but it has been described as present in nearby countries, such as France and Belgium (Rasmont et al. 2017). It is expected that further sampling campaigns will reveal its presence in the country as well.

Since *S. majalis* and *L. pallens* demonstrate particular nesting and foraging behaviour, their apparent rarity might be an artefact of inadequate sampling techniques (Herrmann et al. 2003). However, it cannot be ruled out that local changes in land and biotope management might actually have led to an increase in the number of suitable habitats for these two wild bee species, contributing to their expansion into climatically-favourable areas (Kitt and Reder 2014). For example, it has been hypothesised that the finding of both species in Belgium and the Netherlands (seemingly outside their known geographical range) might correspond to a recent expansion event (Raemakers 2004). The species may have entered the region from the south-west (through France), in a dispersal pattern that excludes the Rhine Valley (Raemakers 2004). Nevertheless, this hypothesis is challenged by findings of both species in the German Federal State of Rhineland-Palatinate (Kitt and Reder 2014) and even at locations as far north as the Botanical Garden of the University of Potsdam (Streese 2020). Future investigations could determine whether the finding of *S. majalis* in Luxembourg supports any of these hypotheses regarding potential dispersal routes and mechanisms, but more specimens would be needed to provide proper arguments for this debate.

### Natural history collections: opportunities and challenges

Our results highlight the importance of NHCs as sources for discoveries and critical re-interpretations of scientific knowledge. In combination with recent fieldwork material, four new wild bee species (*A. lagopus*, *H. papaveris*, *N. furva* and *S. majalis*) were added to the national checklist of Luxembourg. Additionally, we found evidence that supports the current presence in the country of a fifth species (*N. sexfasciata)*, which was omitted during past inventories. With the addition of these findings, the number of wild bee species registered in Luxembourg has increased to 346. The wild bee collection curated at the National Museum of Natural History Luxembourg is preserved under suitable conditions, which will allow future generations of researchers to use, re-examine and debate it in order to answer scientific questions pertinent to their own historical time. As such, our results represent just a small fraction of the exceptional value that NHCs have as repositories for the documentation of the bio- and geodiversity of the world. Despite this great potential, the current decrease in the available funds to keep and curate NCHs threatens the future of several collections, especially the smaller ones, affecting our possibilities to continue profiting from them in the years to come. Therefore, support from the scientific community and funding bodies is imperative, so biological collections can keep growing, documenting and ultimately, fulfilling their role in society.

## Supplementary Material

56E8A31C-F921-5ECC-B527-23B07AE773E5 10.3897/BDJ.9.e64027.suppl1 Supplementary material 1 Updated checklist of Luxembourgish wild bees (346 species) Data type Species list File: oo_530987.xlsx https://binary.pensoft.net/file/530987 Fernanda Herrera-Mesías, Alexander Weigand

71958FD8-B10A-5BD2-A63F-E05C3E4550B2 10.3897/BDJ.9.e64027.suppl2 Supplementary material 2 Additional species suggested by the westpalbees database for Luxembourg. Data type taxonomic File: oo_532963.xlsx https://binary.pensoft.net/file/532963 Alexander Weigand, Fernanda Herrera-Mesías

XML Treatment for Nomada furva

XML Treatment for Hoplitis papaveris

XML Treatment for Nomada sexfasciata

XML Treatment for Andrena lagopus

XML Treatment for Sphecodes majalis

## Figures and Tables

**Table 1.** Taxonomic keys used for the morphological identification of wild bees to species level.

Authors	Title	Used on	Citation
Amiet F, Müller A, Neumeyer R	Fauna Helvetica 9. Apidae 2	* Colletes *	Amiet et al. 1999
Amiet F, Herrmann M, Müller A, Neumeyer R	Fauna Helvetica 9. Apidae 4	Hoplitis*	Amiet et al. 2004
Amiet F, Herrmann M, Müller A, Neumeyer R	Fauna Helvetica 20. Apidae 5	* Nomada *	Amiet et al. 2007
Amiet F, Herrmann M, Müller A, Neumeyer R	Fauna Helvetica 26. Apidae 6	* Andrena *	Amiet et al. 2010
Bogusch P, Straka J	Review and identification of the cuckoo bees of central Europe (Hymenoptera: Halictidae: *Sphecodes*)	* Sphecodes *	Bogusch and Straka 2012
Falk S, Lewington R	Field guide to the bees of Great Britain and Ireland	* Colletes *	Falk and Lewington 2015
Pauly A	Clés Illustrées Pour L’identification des Abeilles de Belgique et des Régions Limitrophes (Hymenoptera: Apoidae) II. Megachilidae	Hoplitis*	Pauly 2015
Dathe HH, Scheuchl E, Ockermüller E	Illustrierte Bestimmungstabelle für die Arten der Gattung *Hylaeus* F. (Maskenbienen) in Deutschland, Österreich und der Schweiz	* Hylaeus *	Dathe et al. 2016
Smit J	Identification key to the European species of the bee genus *Nomada* Scopoli, 1770 (Hymenoptera: Apidae), including 23 new species	* Nomada *	Smit 2018

*The key uses the synonym *Osmia papaveris* (Latreille, 1799) to refer to *Hoplitis papaveris* (Latreille, 1799)
